# The Recognition of Collagen and Triple-helical Toolkit Peptides by MMP-13: SEQUENCE SPECIFICITY FOR BINDING AND CLEAVAGE*

**DOI:** 10.1074/jbc.M114.583443

**Published:** 2014-07-09

**Authors:** Joanna-Marie Howes, Dominique Bihan, David A. Slatter, Samir W. Hamaia, Len C. Packman, Vera Knauper, Robert Visse, Richard W. Farndale

**Affiliations:** From the ‡ Department of Biochemistry, University of Cambridge, Downing Site, Cambridge CB2 1QW, United Kingdom,; the § Cardiff University Dental School, Dental Drive, Cardiff CF14 4XY, United Kingdom, and; the ¶ Kennedy Institute of Rheumatology, Hammersmith, London W6 8LH, United Kingdom

**Keywords:** Collagen, Matrix Metalloproteinase (MMP), Protease, Protein Conformation, Substrate Specificity, MMP-13, Toolkit Peptides, Collagenolysis, Hemopexin Domain

## Abstract

Remodeling of collagen by matrix metalloproteinases (MMPs) is crucial to tissue homeostasis and repair. MMP-13 is a collagenase with a substrate preference for collagen II over collagens I and III. It recognizes a specific, well-known site in the tropocollagen molecule where its binding locally perturbs the triple helix, allowing the catalytic domain of the active enzyme to cleave the collagen α chains sequentially, at Gly^775^–Leu^776^ in collagen II. However, the specific residues upon which collagen recognition depends within and surrounding this locus have not been systematically mapped. Using our triple-helical peptide Collagen Toolkit libraries in solid-phase binding assays, we found that MMP-13 shows little affinity for Collagen Toolkit III, but binds selectively to two triple-helical peptides of Toolkit II. We have identified the residues required for the adhesion of both proMMP-13 and MMP-13 to one of these, Toolkit peptide II-44, which contains the canonical collagenase cleavage site. MMP-13 was unable to bind to a linear peptide of the same sequence as II-44. We also discovered a second binding site near the N terminus of collagen II (starting at helix residue 127) in Toolkit peptide II-8. The pattern of binding of the free hemopexin domain of MMP-13 was similar to that of the full-length enzyme, but the free catalytic subunit bound none of our peptides. The susceptibility of Toolkit peptides to proteolysis in solution was independent of the very specific recognition of immobilized peptides by MMP-13; the enzyme proved able to cleave a range of dissolved collagen peptides.

## Teaser

**Background:** MMP-13 recognizes poorly-defined sequences in collagens.

**Results:** MMP-13 binds key residues in the canonical cleavage site and another site near the collagen N terminus.

**Conclusion:** MMP-1 and MMP-13 differ in their recognition and cleavage of collagen, which is regulated primarily through the Hpx domain of MMP-13.

**Significance:** Our data explain the preference of MMP-13 for collagen II.

## Introduction

Collagens are comprised of three α chains containing repeating Gly-X-X′ triplets (where X and X′ are often Pro and Hyp, respectively). This primary structure allows the formation of a right-handed collagen superhelix, which endows the molecule with resistance to degradation by most proteases. The fibrillar collagens I, II, and III contain a conserved triple-helical COL domain of 1014 residues, with short, non-helical extensions at each end, which constitutes a tropocollagen molecule. Such collagens assemble side-by-side to form a three-dimensional staggered array (a fibril) with a regular offset of 234 residues (one D-period) between adjacent tropocollagens. This organization gives rise to the striated structure of the collagen fiber observed by transmission electron microscopy, where the 67 nm periodicity reflects the dimensions of one D-period (1).

Collagenolytic MMPs are members of the zinc-dependent peptidase family whose tightly regulated proteolytic activities play a pivotal role in extracellular matrix homeostasis, cell migration and wound healing. Under pathological conditions, uncontrolled tissue remodeling by collagenases such as MMP-13 is associated with the potentiation of tumor progression and metastasis, atherosclerotic plaque remodeling and arthritic disease (2). The preferred substrate for MMP-13 is collagen II which is cleaved five times faster than collagen I and six times faster than collagen III (3), and more readily by MMP-13 than by other collagenases.

As with other MMPs, collagenases are secreted as pro-enzymes with an organized domain structure comprising a pro-peptide region (cleaved to yield the active mature enzyme), a globular, Zn^2+^-binding catalytic domain (Cat),^2^ a linker region and a C-terminal 4-bladed β-propeller hemopexin-like domain (Hpx) (4, 5). The Cat and Hpx are thought to cooperate to recognize and bind to the cleavage site, in collagen D-period 4 (D4), three-quarters of the way along the tropocollagen molecule (6, 7). Binding of the Hpx is considered to facilitate local relaxation of the helix, allowing the Cat domain of the MMP to hydrolyze specific Gly-X bonds within Gly-X-X′, where X is either Leu or Ile, and X′ is either Ala or Leu (*i.e.* within the triplet G-[I/L]-[A/L]) (8). Deletion of the Hpx domain therefore results in a loss of collagenase activity (9, 10). To date, two cleavage sites have been identified for MMP-13 within collagen II. The first at Gly^775^–Leu^776^ (numbering refers to position within collagen II helical domain) is shared by MMPs-1 and -8; the second site, Gly^778^–Gln^779^ (11) is three amino acids from the N terminus of the newly-cleaved quarter fragment (12). Several G-[I/L]-[A/L] triplets are present in native collagen, indicating that, in principle, other scissile bonds may exist. However, with the exception of MMP-13, most MMPs primarily cleave the collagen helix at a single location, reflecting the importance of the unique sequence which surrounds the cleavage site (13, 14).

To simplify structural research, a mutant species, MMP-13(E204A), has been produced. The mutation lies within the active site, and MMP-13(E204A) lacks catalytic activity while retaining the same conformation. The specific collagen residues required for recognition of the collagen triple helix and the residues within/surrounding the canonical collagenase site described above have not yet been systematically identified, and with this in view, we have investigated both wild type MMP-13 and MMP-13(E204A) in the present study. The use of both wild type and mutant active forms, together with their corresponding pro-forms and a free Hpx domain allows us to investigate the contribution to binding of all three component domains.

To facilitate mapping studies we synthesized a library of overlapping homotrimeric host-guest peptides, in which 27 residues of primary collagen (guest) sequence is placed between [GPP]_5_ hosts that ensure triple-helical conformation. The last nine guest amino acids are the same as the first nine amino acids in each successive peptide, and these peptide libraries (referred to as Collagen II and III Toolkits, respectively) encompass the entire triple helical domains of collagens II and III (15, 16), reviewed by Farndale *et al.* (17). Here, we used these Toolkits to map MMP-13 binding to collagens II and III, and we proceeded to synthesize subsidiary peptides to identify those residues surrounding the cleavage site that are required for the binding of MMP-13. This systematic approach has also allowed us to identify a new MMP-13 binding site on collagen type II.

The recent elucidation (7) of the structure of MMP-1 in complex with a triple-helical peptide derived from the use of the Toolkits allows us to compare the binding activity of both collagenases to their common cleavage site, and to show that there are marked differences between the binding activities of the two enzymes that may account for their differing specificities for collagens I, II, and III.

## EXPERIMENTAL PROCEDURES

### 

#### 

##### MMP-13 Expression, Purification, and Activation

Recombinant proMMP-13 was expressed and purified as previously described (18). Where required, proMMP-13 was activated to yield MMP-13 by incubation in 1 mm 4-amino-phenylmercuric acetate (APMA) for 1 h at 37 °C. ProMMP-13(E204A) was expressed in *Escherichia coli* BL21(DE3), refolded, purified, and activated essentially as described for proMMP-1(E200A) (19, 20). The Cat domain of MMP-13 (Δ249–451) was expressed and purified from NSO mouse myeloma cells as previously described (12). MMP-13 GST-Hpx domain was expressed in *E. coli* using the pGEX-2T expression vector, the forward primer TCCGCGTGGATCCCTCTATGGTCCAGGAGATGAA and the reverse primer GCAA-ATTCCATTTTGTGGTGTTGAAGAATTCAT, which contain BamHI and EcoRI restriction sites, respectively, as previously described(16).

##### Peptide Synthesis

Collagen Toolkit II and III and other peptides were generated using an AB Systems Pioneer automated synthesizer and *N*-(9-fluorenyl)methoxycarbonyl (Fmoc) chemistry as previously described (15, 16). All peptides were verified using MALDI-TOF mass spectrometry and their triple-helical conformation confirmed by polarimetry.

##### Peptide Design

Unless stated otherwise, all peptides were triple-helical, a structure maintained by the flanking sequences, GPC(GPP)_5_- and -(GPP)_5_-GPC-amide, at their N and C terminus, respectively. For simplicity, peptides are referred to by their specific guest sequence. A negative control peptide, (GPC-(GPP)_10_-GPC-amide) is referred to as GPP_10_. A linear version of Toolkit peptide II-44 contained the same guest sequence between disordered flanking host sequences, thus: GCPP(GPPP)_2_GGPPPG-II-44 guest sequence -P(GPPP)_2_GGPPPGCPG-amide.

##### MMP-13 Toolkit Solid-Phase Binding Assays (SPBA)

Immulon 2 HB 96-well plates (Nunc, Langenselbold, Germany) were coated with Collagen Toolkit or other peptides, fibrillar or monomeric collagen at a saturating concentration (10 μg/ml in 0.01 m acetic acid) overnight at 4 °C. Fibrous bovine type I collagen was a gift from Ethicon Corporation (Somerville, NJ). Monomeric collagen was obtained from Devro (Bathurst, Australia). All further incubations were performed at 24 °C for 1 h unless otherwise stated. The wells were washed three times with adhesion buffer (1 mg/ml BSA in Tris-buffered saline (TBS) containing 0.1% (*v*/*v*) Tween-20) between each incubation step. The wells were blocked with 50 mg/ml BSA in TBS prior to the addition of MMP at a concentration of 83 nm (unless otherwise stated) in adhesion buffer. Where indicated, increasing amounts of Toolkit peptides II-8 and II-44 were pre-incubated for 20 min with the MMP prior to adhesion assays. Rabbit anti-MMP-13, raised against MMP-13 hinge region (Abcam, Cambridge, UK), and goat anti-rabbit HRP (Dako, Ely, UK) were added at a dilution of 1:2000 in adhesion buffer prior to the addition of TMB substrate system (Sigma), and the plates read at 450 nm. Rabbit HRP-linked anti-GST (GE Healthcare; dilution 1:10,000) was used to detect GST-Hpx. To confirm affinity of the anti-hinge antibody for MMP-13 and MMP-13(E204A), increasing concentrations of MMP were coated onto an ELISA plate prior to blocking and detected as previously described. Binding curves were fitted using Prism 5.0 software (GraphPad, San Diego), allowing total binding (*B*_max_) and equilibrium dissociation constant (*K_D_*) to be determined.

##### Biotinylation of MMP-13(E204A) and the Cat Domain

Proteins were C-terminally biotinylated using an EZ-Link® Micro Sulfo-NHS-Biotinylation Kit (Pierce) according to the manufacturer's instructions. Successful biotinylation was detected via Western blotting using an ultrasensitive Streptavidin-Peroxidase Polymer (Sigma).

##### Peptide Digestion Assays

To determine the likelihood of Toolkit peptide clipping by MMP-13 during SPBA experiments, Toolkit peptides at a final concentration of 80 μm were incubated with a high (4.4 μm final) concentration of MMP-13, MMP-13(E204A) or the equivalent volume of Tris buffer pH 7.4 for 1 h at 24 °C. Proteolytic activity of MMP-13 was assessed following incubation of Toolkit peptides with 250 nm MMP for 16 h at 24 and 37 °C, respectively. The samples were then either examined by electrophoresis under reducing conditions, with silver staining, or submitted for MALDI mass spectrometry. SDS-PAGE electrophoresis was performed on 4–12% NuPage® bis-Tris gels (Invitrogen) according to the manufacturer's instructions.

##### Mass Spectrometry Analysis

Peptides in TBS buffer, pH 7.4, were reduced with 5 mm Tris(2-carboxyethyl)phosphine (Thermo Scientific-Pierce) for 10 min at 35 °C and then desalted using uC18ZipTips (Millipore) equilibrated and washed with 5% (*v*/*v*) acetic acid. Peptides were eluted with 2 μl of ferulic acid matrix (Sigma; 10 mg/ml in 50% (*v*/*v*) aqueous acetonitrile), spotted to the MALDI target plate, dried, and washed once with 2 μl of 5% (*v*/*v*) acetic acid. Mass spectra were collected on a MALDI MicroMX Instrument (Waters, UK) in reflectron mode at threshold laser power. Spectra were calibrated externally with polyethylene glycol 1000–2000-3000 (Sigma) and then adjusted by lockmass to one of two known peptides present in all samples from the autodigestion of MMP-13 (20–42 and 21–42, confirmed by *ms*/*ms* as below). Mass accuracies were generally better than 20 ppm, allowing only a single interpretation of the cleavage site for most peptides. Any ambiguities were resolved by ms/ms fragmentation on a separately desalted sample, eluted in 70% MeOH/0.2% (v/v) formic acid, and analyzed on a Thermo LCQ Classic ion-trap instrument using static nanospray delivery. This confirmed the sequence identity.

## RESULTS

### 

#### 

##### Binding of MMP-13 to Toolkit Peptides and Collagen

**FIGURE 1.**
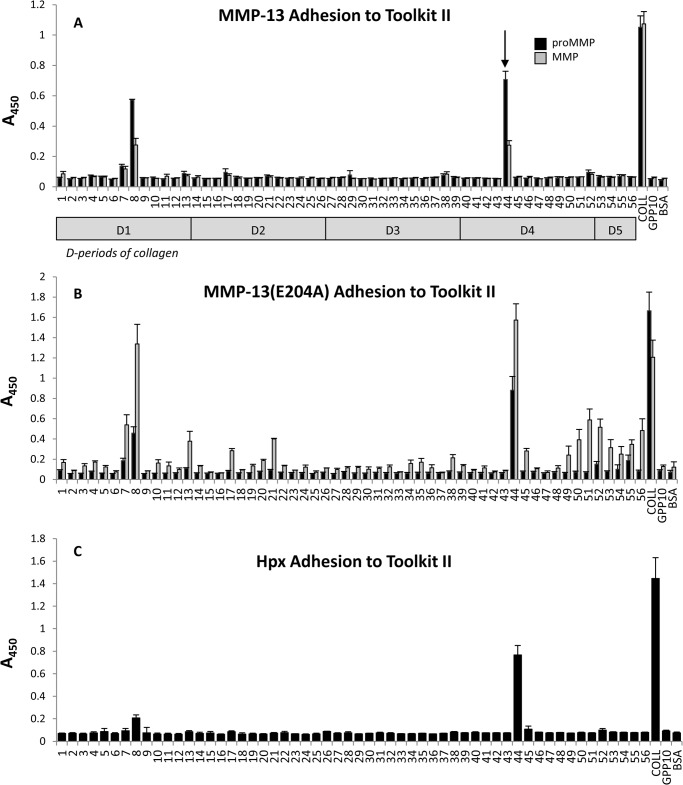
**Adhesion of MMP-13 preparations to Collagen II Toolkit peptides.** Plates were coated with 10 μg/ml Toolkit II peptides, fibrillar type I collagen, and the negative binding peptide GPP_10_. *A*, proMMP-13 (*black bars*) and MMP-13 (*gray bars*). *B*, proMMP-13(E204A; *black bars*) and MMP-13(E204A; *gray bars*). *C*, Hpx at a concentration of 83 nm were allowed to adhere to the peptides for 1 h at 24 °C. Full-length MMP-13 preparations were then detected using an antibody directed at the MMP-13 linker region, and Hpx with an anti-GST antibody as described under “Experimental Procedures.” Data represent mean *A*_450_ ± S.E. of four experiments. For reference, collagen D-periods corresponding to these peptides are shown as *gray bars* below, and the collagenase cleavage site indicated by an *arrow*.

Using SPBA we show that wild type MMP-13, in both its active and pro-enzyme form, bound prominently to just two peptides, II-44 and II-8, with trace binding activity to peptide II-7 (Fig. 1*A*). Binding of MMP-13 to II-44 was slightly stronger than to II-8 with *A*_450_ values of 0.7 and 0.6, respectively. Toolkit III showed uniform absence of binding (data not shown), consistent with the greater activity of the enzyme against collagen II(18). Fibrous collagen I bound wild type MMP-13 strongly (*A*_450_ ≥ 1.0), while control peptides and uncoated wells showed the expected low reactivity (*A*_450_ ≤ 0.1). Using wild type, MMP-13 exhibited lower binding than proMMP-13 to Toolkit peptides.

MMP-13(E204A) and proMMP-13(E204A) also bound Toolkit II, with II-44 and II-8 clearly resolved, again with slight, significant binding to II-7. MMP-13(E204A) was less selective than proMMP-13(E204A), binding several other peptides (II-13, II-17, II-21, II-45, and II-49 to II-56, with *A*_450_ ≥ 0.3; Fig. 1*B*). ProMMP-13(E204A) was constitutively less active overall. This rank order of binding contrasts with that of the wild type enzyme, where the converse applied. MMP-13(E204A), but not proMMP-13(E204A), displayed higher background binding (*A*_450_ ≤ 0.3) on Toolkit III and bound to several peptides, including III-44 that contains the canonical cleavage site, with low to intermediate affinity (*A*_450_ 0.2–0.8; data not shown).

This may suggest that the Cat, which contains the disabling Ala substitution, helps to define binding specificity. Lower binding of proMMP-13(E204A) might suggest that obstruction by the pro-domain restricts access of collagen to the enzyme, diminishing but not abolishing its ability to bind. The lower apparent binding of the active wild type MMP-13 might be due either to inhibition of binding by residual pro-domain peptides, as previously observed (21, 22) or by autolysis, effects that appear to predominate in the active enzyme.

##### Binding Affinity to II-44 and II-8

We applied increasing concentrations of the MMP preparations to peptides II-8 and II-44 to compare binding affinities using SPBA. The linear peptide equivalent to II-44 supported negligible binding of any MMP-13 preparation (data not shown), contrasting with the ability of a similar peptide (23, 24) to support the binding of fibronectin, which recognizes the same site (25, 26). Comparison of the pro-forms of the enzyme on both triple-helical peptides suggests broadly similar affinities (*p* = 0.37), with *K_D_* ranging from 130 to 380 nm, the latter figure (the only outlying value) being the estimate for proMMP-13(E204A) binding to peptide II-8 (data not shown).

**FIGURE 2.**
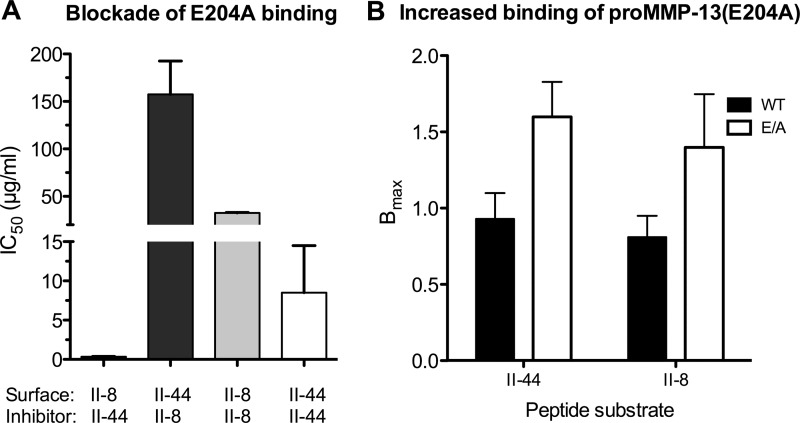
**Competitive inhibition of MMP-13 adhesion to Toolkit peptides II-8 and II-44.** *A*, MMP-13 or MMP-13(E204A) were pre-incubated with increasing amounts of Toolkit peptides II-8 and II-44 prior to adhesion assays on II-8 and II-44, respectively. Absorbance was measured at 450 nm. Data represent the mean ± S.E. of four experiments. *B*, effect of MMP-13(E204A) mutation on increased proMMP-13 adhesion to Toolkit peptides II-8 and II-44. Binding of MMP-13 and MMP-13(E204A) to coated ELISA wells was performed as described in Fig. 1. Both pro- and active forms of the enzyme exhibited broadly similar affinities (*p* = 0.37), with *K_D_* ranging from 130 to 380 nm. Data represent the mean ± S.E. of four experiments.

Our Toolkit and SPBA data indicate that both the pro and active forms of MMP-13 bind more strongly to II-44 than II-8. To investigate the relative affinities of these interactions, we pre-incubated MMP-13(E204A) with II-8 and II-44 ranging from 0.5 to 50 μg/ml and then measured binding in SPBA to the same immobilized peptides. Consistent with the data above, each peptide in solution blocked MMP-13(E204A) adhesion to its own immobilized form (Fig. 2*A*), with II-44 showing IC_50_ ∼8 μg/ml, and II-8 about a 4-fold weaker inhibition. II-8 caused very slight inhibition of adhesion to II-44 at up to 50 μg/ml (extrapolated IC_50_ ∼ 150 μg/ml) while II-44 was a potent inhibitor of binding to II-8 (IC_50_ < 0.5 μg/ml). These data confirm II-44 as a more potent ligand for MMP-13 than II-8 (*p* = 0.017, Kruskal-Wallis test).

It was noted that *B*_max_ was dependent on the presence of the active site mutation, being roughly double in the proMMP-13(E204A) and MMP-13(E204A) mutant forms compared with their wild type counterparts (*p* < 0.02, 2-way ANOVA, Fig. 2*B*). This was not due to different recognition of wild type and MMP-13(E204A) by the detecting antibody (data not shown).

##### Binding to Full-length Collagen I

**FIGURE 3.**
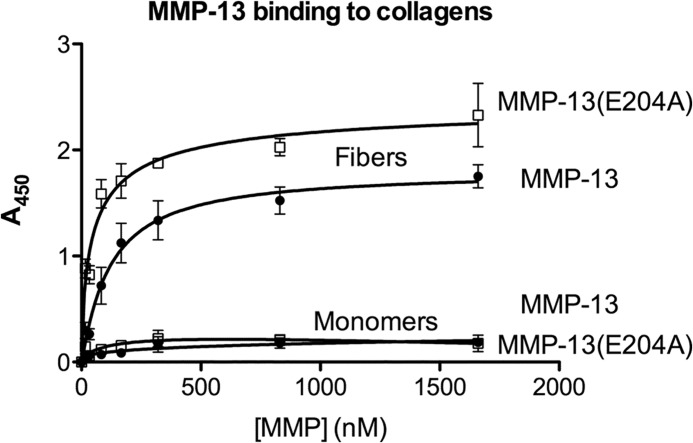
**Binding of MMP-13 and MMP-13(E204A) to monomeric and fibrillar type I collagens.** Binding of MMP-13 and MMP-13(E204A) to collagen-coated ELISA wells was performed as described in Fig. 1. For MMP-13 binding to fibers and monomers, *K_D_* values were 33 and 118 nm, respectively, and for MMP-13(E204A), 52 and 26 nm, respectively. Data points represent the mean ± S.E. of four experiments, and differences were not significant.

Active forms of MMP-13 and MMP-13(E204A) were used in similar assays to establish the ability of the enzyme to bind fibrillar and monomeric collagens (Fig. 3). Both enzyme preparations exhibited much lower binding to saturated coatings of monomeric collagen I than to II-44. This may reflect the lower density of binding sites in monomeric collagen, where there are two sites per 1000 residues, compared with one site per 63 residues in these Toolkit peptides. The two substrates are directly comparable, since rotary shadowing/transmission electron microscopy (27) suggests that monomeric collagens lie flat upon a surface, as would also be anticipated for the much shorter Toolkit peptides.

Our data suggest that the binding of the MMP to collagen is greatly facilitated by fibrillar conformation (*K_D_* ∼40 nm). Pugh *et al.* have shown that collagen fibers as used here form a complex meshwork that extends tens of microns above the coated surface (28), offering a large binding area. The observed higher affinity implies co-operative binding to discrete sites within the fiber network. Possibly, a new composite binding site comprising the canonical site and sequences in the adjacent tropocollagen molecules within a single fiber might also generate a larger footprint on the fiber surface, and hence higher affinity.

##### Binding of Cat and Hpx Toolkit Peptides II-8 and II-44

To determine whether the MMP-13 Cat and Hpx domains were individually able to recognize Toolkit peptides, their binding was assessed in SPBA. Though the Cat domain was able to recognize collagen, it was unable to bind to any Toolkit II peptides (data not shown), while Hpx bound well to peptide II-44 (*A*_450_ ∼0.8) and weakly to II-8 (*A*_450_ ∼0.2; Fig. 1*C*). Both domains bound fibrillar collagen I, albeit weakly in the case of the Cat domain.

##### Ala-scanning of II-44

**FIGURE 4.**
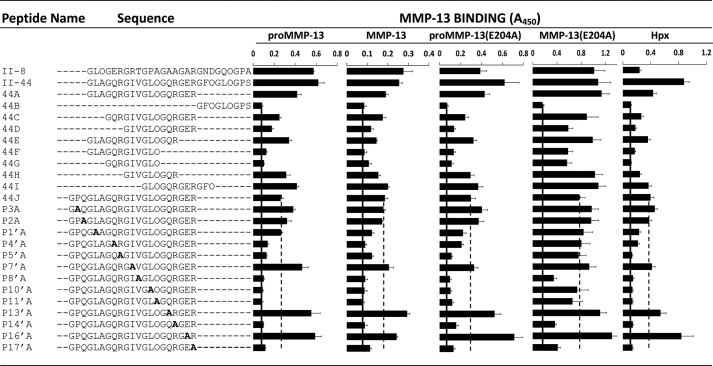
**Adhesion of proMMP-13, MMP-13, proMMP-13(E204A), MMP-13(E204A), and the Hpx domain to II-44 variant Toolkit peptides was carried out as described for Fig. 1.** The *dashed line* represents MMP adhesion level on peptide II-44J, and the *solid line* MMP baseline adhesion on peptide II-44B. Data represent the mean ± S.E. of four experiments.

To explore the primary sequence determinants of MMP-13 binding to II-44 in SPBA, we made a set of truncated and alanine-substituted (Ala-scanned) triple-helical peptides of the same general host-guest form. The data are shown in Fig. 4. Binding of (MMP-13(E204A), and to a lesser extent wild-type MMP preparations, proMMP-13 (E204A), and Hpx was supported by an 18-residue triple-helical peptide, II-44A, which lacked only the three C-terminal triplets of II-44. Peptide 44B, containing only these missing triplets, GFOGLOGPS, supported no binding. Successive omissions of triplets (peptides II-44C to II-44I) from both the N and C terminus of II-44A each modulated the binding of MMP-13(E204A), indicating that further truncation was inappropriate. These findings were used to define the C terminus of the Ala-scanned sequence, culminating in the addition of a single-triplet native sequence extension, GPQ, to the N-terminal boundary of II-44, thus creating a 21-residue peptide (44J). Using the nomenclature of Schechter and Berger to define the distance from the cleavage site, peptide 44J, GPQG∼LAGQRGIVGLOGQRGER, stretched from P_4_ to P_17_′ where P_1_, preceding the scissile bond, is the first (Gly) residue of the guest sequence of peptide II-44.

As with II-44, binding to 44J differed among MMP-13 preparations, with MMP-13(E204A) binding most strongly (*A*_450_ ∼0.8), then proMMP-13, then proMMP-13(E204A), then active MMP-13, and lastly the Hpx domain alone (*A*_450_ ∼0.4). All MMP-13 preparations yielded data that allowed us to discriminate between the Ala-substituted peptides.

Three residues were uniformly essential for binding, P_8_′(V), P_14_′(R) and P_17_′(R). No other Ala-substitutions perturbed the binding of MMP-13(E204A), consistent with less rigorous determinants of peptide binding to this mutant form, but several residues were important for the binding of the other MMP preparations including the Hpx domain. P_4_′(Q), P_5_′(R), P_10_′(L), and P_11_′(O) all fall into this category, while P_1_′(L) was similarly important for the binding of Hpx, and to a lesser extent MMP-13 and proMMP-13(E204A).

Two residues in particular in the parent peptide, P_13_′(Gln) and P_16_′(Glu), appeared to exert a restrictive effect, since MMP-13 binding increased after Ala-substitution. Further residues, P_3_(Pro), P_2_(Gln) and P_7_′(Ile), showed a similar tendency with some but not all MMP-13 preparations. These data suggest that recognition of the native sequence is poised between firm binding and release of the enzyme, perhaps allowing some freedom to re-organize or to permit relaxation of the triple-helical structure prior to cleavage, or to ensure release of the enzyme once the substrate is cleaved.

Like the intact forms of the enzyme, Hpx only bound to Toolkit peptides II-8 and II-44 in the static adhesion assays (Fig. 1*C*). The adhesion profile of the Hpx domain to II-44 Ala-scanned peptides reflected that of the intact enzyme(s), indicating that it is primarily the Hpx domain, which defines the recognition of the intact enzyme to the collagenase cleavage site of collagen II.

##### Proteolysis of Toolkit Peptides

We first considered the possibility that poor binding of active MMP-13 to peptides or collagen might reflect proteolysis and release from the 96-well surface during SPBA, leading to loss of apparent binding activity. Our binding data also suggested that peptide II-8 may offer a novel cleavage site for MMP-13. We therefore investigated the ability of MMP-13 to cleave several triple-helical peptides, selected by their differing ability to bind MMP-13(E204A). Peptides II-44 and II-8 (high-affinity), III-5 and III-40 (intermediate affinity), and II-24 and II-28 (non-binding), were incubated with a high concentration (4.4 μm) MMP-13 for 1 h at 24 °C, then examined using SDS gel electrophoresis as described. The migration of the peptides was slower than their mass would suggest, an observation which is well known. The actual masses of the peptides are shown in Table 1. No proteolysis of any Toolkit peptide was observable after 1 h at 24 °C (data not shown), despite the rather high enzyme concentration used, allowing us to conclude that our binding assays were not compromised by substrate degradation. As expected, MMP-13(E204A) (and buffer control) proved unable to degrade any peptide under any condition tested in the present study.

**TABLE 1:**
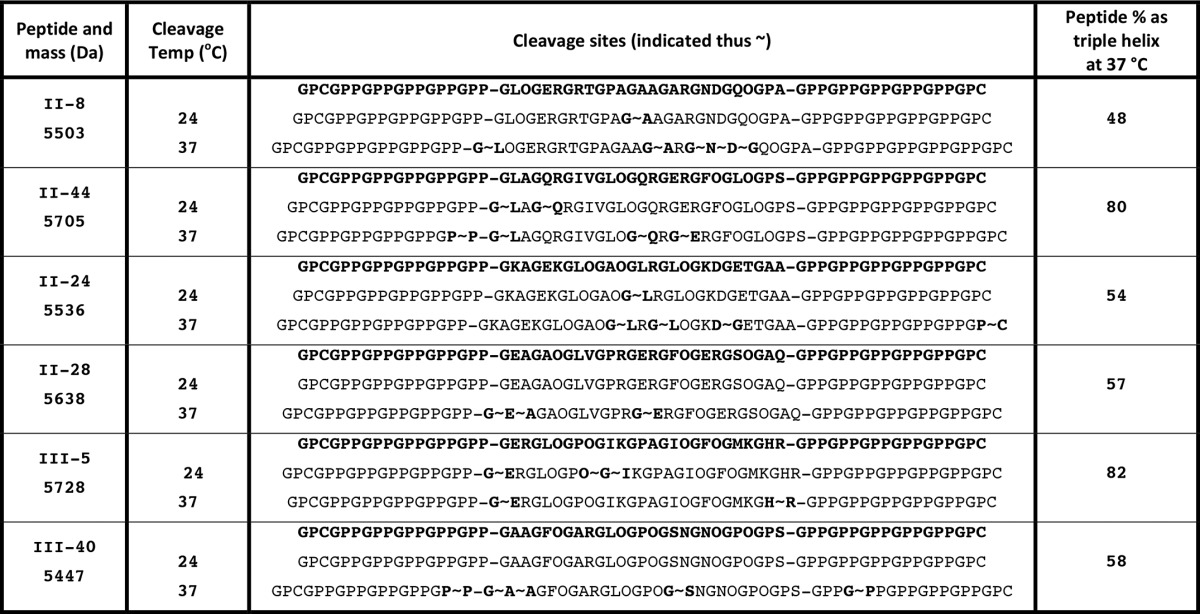
**Mass analysis of degraded Toolkit peptides** Following degradation for 16 h at 24 °C and 37 °C as described for Fig. 9, *B* and *C*, respectively, Toolkit peptides incubated with MMP-13 or Tris buffer were subjected to MALDI mass spectrometric analysis to determine the peptide cleavage site(s) of MMP-13. Cleavage sites are summarized with the site of proteolysis after the first highlighted residue.

**FIGURE 5.**
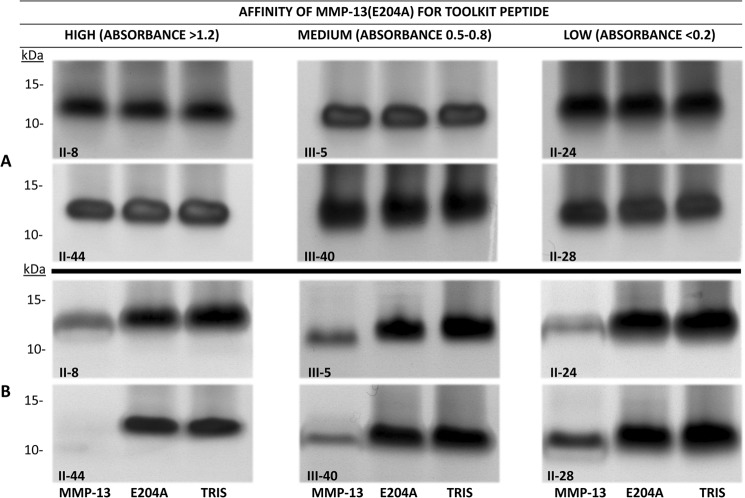
**Analysis of degradation of Toolkit peptides by active MMP-13.** Toolkit peptides at a final concentration of 80 μm to which MMP-13(E204A) displayed high binding (II-8 and II-44; *A*_450_ > 1.2); intermediate binding (Toolkit peptides III-5 and III-40; *A*_450_ 0.5–0.8) and low binding (Toolkit peptides II-24 and II-28; *A*_450_ < 0.2) were incubated with Tris buffer, MMP-13 or MMP-13(E204A) at 250 nm final concentration at (*A*) 24 °C for 16 h and (*B*) 37 °C for 16 h. After incubation, samples were subjected to electrophoresis under reducing conditions and silver stained. Images are representative of three experiments.

However, following incubation of the peptides with 250 nm MMP-13 for 16 h at 24 °C, a temperature at which the majority of each peptide is folded as a triple helix, proteolysis of II-8, II-44, II-24, and III-5 was detectable by mass spectrometry. Cleavage sites are shown in Table 1. Degradation was slight and not detectable using SDS-PAGE (Fig. 5*A*). At 37 °C, proteolysis was clearly observable by SDS-PAGE for all peptides tested (Fig. 5*B*). Cleavage sites were subsequently confirmed by MALDI mass spectrometry (Table 1). At 24 °C, peptide II-44 was specifically cleaved at both its primary and secondary collagenase cleavage sites (G∼L and G∼Q, respectively). Peptide II-8 was cleaved, but at a G∼A bond downstream from the expected G∼L cleavage site. At 37 °C, however, the degradation of II-44 was increasingly marked and less specific, most likely due to the gelatinase activity of MMP-13.

## DISCUSSION

We used four full-length MMP-13 preparations to screen the Toolkit peptides; the wild type pro-form expressed and purified from mouse myeloma cells, and recombinant MMP-13(E204A) and proMMP-13(E204A) expressed in pET3 cells as previously described (18, 20). The active wild type MMP-13 was prepared by APMA-induced cleavage of its pro-form (18, 29). In addition, we tested the binding properties of recombinant Cat and Hpx domains. The present work shows that collagen and two triple-helical Toolkit peptides, II-44 and II-8, support firm adhesion of MMP-13 in both its active and pro- forms, and of the equivalent catalytically inactive mutant forms, proMMP-13(E204A) and MMP-13(E204A). Cat bound weakly to collagen, but not to any Toolkit peptide, while Hpx bound to II-8 and to a greater extent II-44, much like proMMP-13(E204A).

### 

#### 

##### The Fidelity of MMP-13(E204A) Substrate Recognition

The use of MMPs that are alanine-substituted at the catalytic site simplifies structural and binding studies, since both autolysis and digestion of adhesive substrates are avoided. Here, we observe differences in behavior of the active conformation of MMP-13(E204A) compared with either its own pro-form or both the active or pro-forms of the wild type enzyme. We find MMP-13(E204A) to bind less specifically to Toolkits, with extra hits observed in Toolkit II and a noisy pattern of binding to Toolkit III. MMP-13(E204A) was less sensitive to Ala-scanning of peptide II-44, notably at P_5_′, P_10_′, and P_11_′ (Fig. 4). In contrast, proMMP-13(E204A) behaved exactly as pro- or active wild type. We also found a 2-fold higher *B*_max_ for MMP-13(E204A), which implies twice the number of molecules binding per peptide. In turn, this observation suggests dimerization *in situ*, the first immobilized copy of MMP-13(E204A) recruiting a second molecule, a process mediated by interactions remote from the peptide-binding surface of the enzyme. Crystallographic analysis by Stura *et al.* (22) indicates close agreement between the structures of native and mutant forms, but they also report MMP-13(E204A) to be monomeric in solution despite observing dimerization in their crystal structure, mediated by the Hpx domain. Reconciling these findings suggests that the E204A substitution subtly modulates the relationship between the Cat and Hpx domains in the active conformation of the enzyme, in a way that renders the Hpx less selective in recognizing collagenous substrates.

**TABLE 2:** **Summary of the effect of alanine substitution on MMP-13 binding** Residues of Toolkit peptide II-44J are listed along with the residues of MMP-1 that interact with these amino acids. (MMP-1 residues in *bold* are conserved in MMP-13.) The effect of alanine substitution of each residue is annotated as: *ND*, not done; [ ], residue lies upstream of guest sequence in peptide II-44J; –––, ––, reduction of MMP-13 binding to peptide by ≥ 50%, and ≥ 25%, respectively; 0 is no observed change in MMP-13 adhesion to peptide; +, ++, increase in MMP-13 binding of 25–0% and ≥ 25%, respectively.

Position	Residue	Effect of Ala substitution on MMP-13 binding	Residues of MMP-1 in contact with peptide
[P_5_]	[O]	[ND]	[**Phe^166^]**
P_4_	G	ND	
P_3_	P	0	Asn^152^, Gln^167^
P_2_	Q	0	Ser^153^, **Ala^163^, His^164^**
P_1_	G	ND	Asn^161^
P_1_′	L*^a^*	**– –**	**Ala^165^, His^203^**
P_2_′	A	ND	**Gly^160^, His^209^**
P_3_′	G	ND	
P_4_′	Q	**–––**	Asn^161^, Tyr^218^, Ser^220^, **Tyr^221^**
P_5_′	R	**– –**	Tyr^218^, **Pro^219^, Ser^220^**
P_6_′	G	ND	
P_7_′	I	0	**Glu^294^, Asn^296^, Val^300^, Phe^301^**
P_8_′	V	**–––**	**Arg^285^, Val^300^, Phe^301^**
P_9_′	G	ND	**Arg^285^**
P_10_′	L*^a^*	**–––**	Ile^271^, **Glu^274^, Met^276^, Arg^285^**, Phe^289^, Tyr^290^, **Phe^301^, Trp^302^**
P_11_′	O	**–––**	**Arg^272^, Glu^274^, Gln^335^**
P_12_′	G	ND	
P_13_′	Q	++	
P_14_′	R	**–––**	
P_15_′	G	ND	
P_16_′	E	+	
P_17_′	R	**– –**	

*^a^* Ala substitution that reduced MMP-1 adhesion relative to II-44J.

##### Collagen Residues Critical for Recognition by MMP-13

**FIGURE 6.**
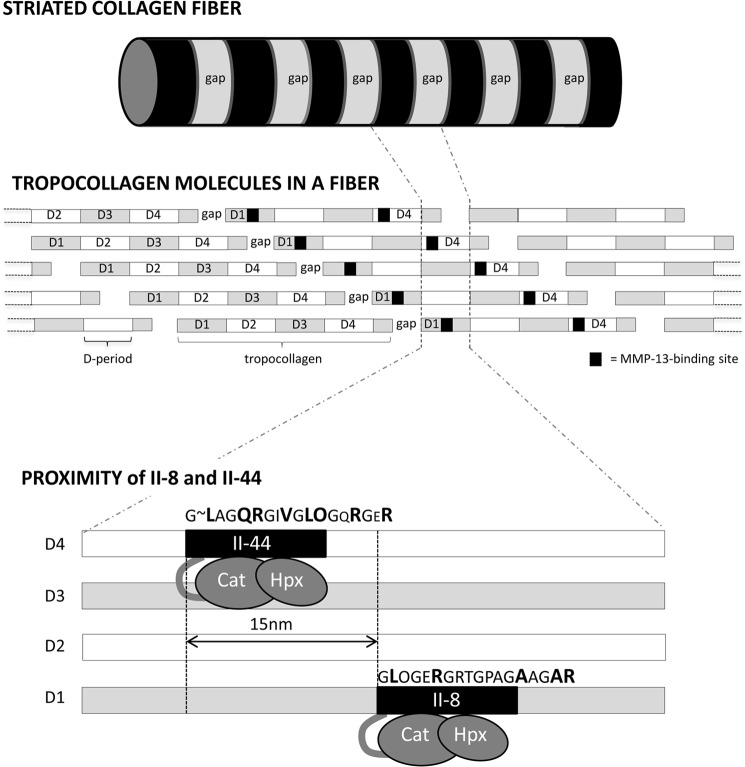
**Schematic illustration of a collagen fiber.** *Above* shows the alternating gap (*pale*) and overlap (*dark*) banding, derived from the staggered assembly of the fiber from tropocollagen molecules. *Middle,* the “quarter stagger” between tropocollagen molecules is shown, with labeled D-periods alternating (*gray* and *white*) with the MMP-13 binding sites mapped onto them (*black*). *Below* the linear separation between the II-44 and II-8 sites is shown, and their sequences provided. Key binding residues in II-44 and II-8 are *larger, bold font*, while restrictive residues (II-44) are in the *smaller font*. Lateral separation between the axes of the tropcollagen monomers (not indicated) is of the order of 3 nm.

Binding of MMP-13 to peptide II-44 was expected, since it contains the known collagenase cleavage site near the N terminus of collagen D-period 4, whereas the prominent but lower affinity of MMP-13 for II-8 was unsuspected; its sequence occurring in the center of D-period 1 (see Fig. 6). Equilibrium binding studies suggest similar affinities of all the MMP forms tested for II-44, with *K_D_* on the order of 200–400 nm. Ala-scanning of 44J, a shorter peptide derived from II-44, revealed several residues that make important contributions to the binding of MMP-13, *i.e.* that Ala-substitution diminishes binding. Five of these lie within the C-terminal half of the guest sequence of the peptide, P_8_′(V), P_10_′(L), P_11_′(O), P_14_′(R), and P_17_′(R). Conversely, substitution of two further residues in this region P_13_′(Q) and P_16_′(E) enhanced the binding of all MMP-13 preparations. These observations underline the importance of this tract of the peptide in securing MMP-13 binding, and clarify the basis for its interaction with peptide II-8. II-8 shares just three critical residues with II-44, corresponding to P_1_′(L), P_5_′(R) and P_17_′(R), but in addition, contains two alanine residues, corresponding to P_13_′ and P_16_′. Ala-substitution of both residues in peptide II-44J enhanced MMP-13 binding. Good binding of II-8, especially in the region predicted to bind Hpx, might therefore be anticipated.

##### The Importance of Hpx in MMP-Collagen Interaction

The distance of many of these residues from P1∼P1′ suggests that the Hpx domain is the main contributor to MMP-13 binding, investigated further using isolated Hpx, which bound to both II-8 and II-44, exactly echoing the adhesion profile of proMMP-13(E204A) on the Ala-scanned peptide set (Fig. 4). These data suggest a second site of interaction for Hpx in II-44. In the first, “productive” mode of binding, Hpx engages residues P_10_′ to P_17_′, G**LO**GQ**R**GE**R**, which would align the scissile P_1_∼P_1_′ (G∼L) bond close to the catalytic site. The inhibition of Hpx binding by Ala-scanning at P_1_′, P_4_′, and P_5_′ indicates that these conserved residues, G∼**L**AG**QR**GIV, can also interact directly with the Hpx domain, offering a second locus that can recruit MMP-13 to the environs of its site of action. In this case however, alignment of the Cat with P_10_–P_9_ would not be productive, as the proline-rich collagen sequence at this location would restrict local unwinding by the MMP-13 enzyme.

##### Comparison of MMP-13 with MMP-1

Our interpretation is informed by the crystal structure of the complex between MMP-1 and a triple-helical peptide derived from II-44J. Inspection of the MMP-1-peptide co-crystal shows that P_8_′ and P_10_′ interact with the proximal rim of the Hpx β-propeller, binding to the interface between blades 1 and 2, with the distal rim of Hpx tilted away from the axis of the peptide. No interactions were observed beyond P_11_′. Only two peptide residues, P_1_′(L) and P_10_′(L), had measurable effects on binding of MMP-1 using a very similar SPBA, while effects of substitutions distal to P_10_′ were negligible. In contrast, using MMP-13, there were obvious effects of Ala-substitutions from P_10_′(L) to P_17_′(R), suggesting that its Hpx enjoys a closer interaction with regions of the peptide extending further away from the scissile G∼L bond.

Another marked difference between the two enzymes is that proMMP-1 did not bind to collagen or Toolkit peptides, with the pro-domain apparently impeding access to the Cat (7, 31), preventing the critical P_1_′ interaction with substrate. How can MMP-13 avoid this constraint? Superposition of the Cat domains from the crystal structures of full-length MMP-1 and MMP-13 by Stura *et al.* illustrates the different Cat-Hpx interface in the two enzymes. The two domains move closer together in MMP-13, with Hpx rotated relative to Cat by ∼30°. The effect is to move the collagen-binding S_10_′ pocket of MMP-13 by about 7 Å relative to that of MMP-1(22). This might facilitate collagen-proMMP-13 binding without displacement of pro-domain from the S_1_′ site. Like MMP-13, both proMMP-3 and proMMP-9 can bind collagen (32). Alternatively, although activation of MMPs is achieved through proteolytic cleavage, interaction with substrate may also displace the pro-domain from the active center of the enzyme (33). Nonetheless, our SPBA data suggests that the pro-domain does impede collagen binding to some degree: while the two full-length pro-forms of MMP-13 showed similar binding, the catalytically dead MMP-13(E204A), lacking pro-domain, showed highest binding to Toolkit peptides.

##### The Significance of the II-8 Binding Site

The presence of this site was not expected, and its potential as a substrate for the collagenase activity of MMP-13 appears restricted to the G∼A clip observed at 24 °C. It is located at the center of II-8, four triplets C-terminal to the more promising GLOGER motif that aligns well with the canonical site in II-44; both sites occur at the start of the guest sequence in their respective peptides, and in nature, P_4_ to P_1_ are GPRG and GPQG, respectively. Q, preceding the canonical (II-44) site, is well represented at P_2_ in the MEROPS database (34), while R, preceding the optimally aligned II-8 site, occurs just 4 times in 41 observations of MMP-13 activity against collagenous substrates. This locus in II-8 was clipped at 37 °C, and should not be discounted as an authentic site for the collagenolytic activity of MMP-13.

The two sites may cooperate in recruiting MMP-13 to collagen. In 2007, Overall and Butler (21) proposed an “inchworm” model of MMP translation along the collagen molecule, based on the “burnt bridges” molecular ratchet model (for MMP-1) of Saffarian *et al.* (40), and the atomic force study of Rosenblum *et al.* (41) on MMP-9. For this model to be generally applicable, the MMPs would need to have affinity for long stretches of sequence within the collagen helix, as well as the capacity for sequence-independent cleavage of triple-helical collagen. Manka *et al.* (7) found that MMP-1 lacked the ability to bind well to any site other than II-44 in collagen II, and we show here that MMP-13 is only slightly less selective, binding to II-8 as well as to the second site in II-44. The “inchworm” model may therefore have limited validity for MMP-13, applying to translocation of Hpx binding from the P_1_′ to P_5_′ tract to P_10_′ to P_17_′. The “quarter-stagger” assembly of the collagen fiber dictates that any D-period is aligned side by side with all others (see Fig. 6). Thus, a molecule that bound at the start of D1 would be quite close to the start of D2, D3, D4, and of the truncated D5 (the gap region) in adjacent tropocollagen molecules. The simple model of fiber assembly allows us to calculate the proximity of any two sites. GLAGQR in II-44 represents residues 775–780 of the tropocollagen helix, or 73–78 of the D-period, whereas GLOGER in II-8 is residues 127–132 of both the helix and D-period. Within a fiber, therefore, there is an axial offset of 54 residues between the two sites, corresponding to about 15 nm, close enough, perhaps, to support some co-operativity. Inspection of the co-crystal of MMP-1 and a II-44-derived triple-helical peptide (7) suggests that an MMP can span about 25 collagen helix residues, less than 10 nm. Although not contiguous, therefore, it is plausible that hydrolysis of the canonical site might lead to anchorage of MMP-13 by its Hpx domain to the clipped helix, with sufficient flexibility in the clipped collagen to relocate via its Cat to nearby II-8 sites. This “ball and chain” model would also apply to adjacent canonical sites in D4. The three-dimensional structure may be more complex than the simple model (35, 36), but in either of these two, reciprocal, renderings of the fiber, hydrolysis at one site could reveal the other site beneath.

Finally, whether it is a cleavage site or not, it is plausible that II-8 fulfils a depot function, acting to sequester proMMP-13 in the collagen matrix to be activated as required. MMP-13 differs from MMP-1 in this respect, in that only the active form of MMP-1 was found to bind to Toolkit II (7).

##### The Selectivity of MMP-13 for Different Collagens

Comparison of the collagenase cleavage sites of the various fibrillar collagens with the sequences of the MMP-13-binding peptides provides a basis for their relative recognition and subsequent cleavage. High identity occurs between α1(I) and α1(II), with only two non-identical but conserved amino acids within the sequence equivalent to II-44. The reported 6-fold lower activity of MMP-13 on collagen I may therefore reside in the different sequence at P_4_′ and P_5_′ in α2(I), where A and O occur in place of Q and R, both of which, when replaced with A in peptide II-44J, support reduced binding of active MMP-13. In collagen III, only three (P_5_′(R), P_11_′(O) and P_17_′(R)) of the eight critical amino acids are identical with those in collagen II. We therefore anticipate a lower affinity interaction of MMP-13 with collagen III, consistent with its relative resistance to proteolysis.

##### Toolkit Peptides as Substrates for Proteolysis by MMP-13

We tested the ability of MMP-13 to digest six peptides during 16 h at either 24 °C or 37 °C by examining peptide fragments by mass spectrometry. After incubation at 24 °C, we observed both expected clips in II-44, at P_1_∼P_1_′ and P_3_′∼P_4_′, at the canonical and secondary sites that are well-known(12). At this temperature, only five other clips were located in the six peptides tested, including a G∼A bond in II-8. We had anticipated that the G∼L bond at the start of the guest sequence of II-8, equivalent to P_1_∼P_1_′ in II-44, might be susceptible, best aligning with the catalytic site according to our interpretation of Ala-scan data, but it proved otherwise. The MEROPS database (34) describes the preferred MMP-13 cleavage sequence as being G**P**xG∼LxGx, with proline (bold) occurring at P_3_ in 92 of 147 sites, P being the most abundant P_3_ residue in both collagenous and non-collagenous substrates. The sequence cleaved in II-8 conforms well, the site being GPAG∼AA, with no other imino acids occurring within 9 residues, which may contribute to a more relaxed structure that facilitates proteolysis (37).

At 37 °C, all Toolkit peptides tested were cleaved by MMP-13, irrespective of their ability to bind MMP-13(E204A) in SPBA, and 20 different sites were identified in addition to the 7 found at 24 °C. We found four cleaved G∼L sites, from a total of ten occurrences of GL in the six peptides tested. Five of these ten were within G∼LO triplets, only one of which was cleaved, suggesting that the hydroxyproline at P_2_′ confers a protective effect, which might account for the resistance of the GL bond in II-8 to hydrolysis. We also found four G∼E sites, of only eight GEx′ triplets within the peptides tested. Deng *et al.* report that the hydrophobic MMP-13 S_3_ subsite has a preference for Pro in P_3_ (38), and propose a consensus motif, PLG∼MR. Our limited data suggest a novel consensus sequence: PPG∼ER, with POG∼AX′ and POG∼LA as alternatives. Our data concur with that compiled in MEROPS, although G∼E cleavage is only recorded twice therein, as proline occurs as P_3_ in 15 of the 27 cleavage sites (∼55%) we report here, regardless of temperature.

We considered why elevating temperature might increase the number of resolvable cleavage sites. Firstly, reaction rates will increase, but secondly, our peptides have limited thermal stability, with melting temperatures typically of 45 °C. Thus at 37 °C a substantial proportion (18–52%), estimated for each specific peptide and shown in Table 1, exists in unfolded, single-stranded form (39) where, unlike native collagens, tertiary structure no longer governs substrate specificity and MMP-13 functions as a gelatinase (30). Digestion of any single-stranded material that is susceptible to proteolysis by MMP-13 will draw the equilibrium in favor of unfolded peptide, leading to gradual depletion of intact triple helix. Mass spectrometry revealed that few of the common peptide fragments were generated by cleavage within the GPP hosts. This may reflect the greater thermal stability of the relatively proline-rich host sequence, which unfolds less readily and so presents a less frequent target for proteolysis by MMP-13 than the guest sequence.

In summary, here we show that, unlike MMP-1, both the pro- and active forms of MMP-13 are able to bind collagen, at both the canonical MMP cleavage site, and at a second, lower-affinity site located in Toolkit peptide II-8 (corresponding to D-period 1 of the collagen monomer). We show that the recognition of II-44 by MMP-13 is primarily mediated by the Hpx domain and is specified by GL*X*GQR motifs at either P_1_-P_5_′ or P_9_′-P_14_′. By examining the cleavage of selected collagen peptides by MMP-13, we show that hydrolysis is not confined to motifs recognized by the Hpx domain, but is likely to include peptide strands that are unfolded at 37 °C. These data provide a rationale for the structural regulation of collagen proteolysis and the resistance of peptide II-8 to cleavage by MMP-13 at 24 °C.

## Footnotes

2 The abbreviations used are:

Cat: catalytic domain
MMP: matrix metalloproteinase
Hpx: hemopexin-like domain.
